# Phylogeography and genetic diversity of a widespread Old World butterfly, *Lampides boeticus *(Lepidoptera: Lycaenidae)

**DOI:** 10.1186/1471-2148-8-301

**Published:** 2008-10-30

**Authors:** David J Lohman, Djunijanti Peggie, Naomi E Pierce, Rudolf Meier

**Affiliations:** 1 Department of Biological Sciences, National University of Singapore, 14 Science Drive 4, Singapore 117543, Republic of Singapore; 2 Division of Zoology, Research Centre for Biology-LIPI, Jl. Raya Jakarta-Bogor Km. 46, Cibinong-Bogor 16911, Indonesia; 3 Museum of Comparative Zoology, Harvard University, 26 Oxford St., Cambridge, Massachusetts 02138, USA

## Abstract

**Background:**

Evolutionary genetics provides a rich theoretical framework for empirical studies of phylogeography. Investigations of intraspecific genetic variation can uncover new putative species while allowing inference into the evolutionary origin and history of extant populations. With a distribution on four continents ranging throughout most of the Old World, *Lampides boeticus *(Lepidoptera: Lycaenidae) is one of the most widely distributed species of butterfly. It is placed in a monotypic genus with no commonly accepted subspecies. Here, we investigate the demographic history and taxonomic status of this widespread species, and screen for the presence or absence of the bacterial endosymbiont *Wolbachia*.

**Results:**

We performed phylogenetic, population genetic, and phylogeographic analyses using 1799 bp of mitochondrial sequence data from 57 specimens collected throughout the species' range. Most of the samples (>90%) were nearly genetically identical, with uncorrected pairwise sequence differences of 0 – 0.5% across geographic distances > 9,000 km. However, five samples from central Thailand, Madagascar, northern Australia and the Moluccas formed two divergent clades differing from the majority of samples by uncorrected pairwise distances ranging from 1.79 – 2.21%. Phylogenetic analyses suggest that *L. boeticus *is almost certainly monophyletic, with all sampled genes coalescing well after the divergence from three closely related taxa included for outgroup comparisons. Analyses of molecular diversity indicate that most *L. boeticus *individuals in extant populations are descended from one or two relatively recent population bottlenecks.

**Conclusion:**

The combined analyses suggest a scenario in which the most recent common ancestor of *L. boeticus *and its sister taxon lived in the African region approximately 7 Mya; extant lineages of *L. boeticus *began spreading throughout the Old World at least 1.5 Mya. More recently, expansion after population bottlenecks approximately 1.4 Mya seem to have displaced most of the ancestral polymorphism throughout its range, though at least two early-branching lineages still persist. One of these lineages, in northern Australia and the Moluccas, may have experienced accelerated differentiation due to infection with the bacterial endosymbiont *Wolbachia*, which affects reproduction. Examination of a haplotype network suggests that Australia has been colonized by the species several times. While there is little evidence for the existence of morphologically cryptic species, these results suggest a complex history affected by repeated dispersal events.

## Background

The study of speciation lies at the nexus of micro- and macroevolution, *i.e*., phylogenetics and population genetics. Phylogeography, which incorporates both approaches in a geographical context, examines the role of different historical processes in population demography, differentiation and speciation [1]. The advent of rapid and affordable DNA sequencing over the past 15 years has catalyzed studies on the evolutionary dynamics of populations and the discovery of previously unrecognized morphologically cryptic species [2].

The pea blue butterfly, *Lampides boeticus *(L.) (Lepidoptera: Lycaenidae), is one of the most widely distributed butterflies in the world, and is currently found across the Palaearctic region from Britain to Japan, throughout suitable habitat in Africa, Madagascar, South East Asia, and Australia, extending eastwards to parts of Oceania including Hawaii. It occurs in temperate, subtropical, and tropical biomes in both lowland and montane localities, typically in open and/or disturbed areas.

Taxonomically, *L. boeticus *is the only species in its genus and has no commonly recognized subspecies, despite its wide distribution. The larval stages feed on plants in at least six families, although Leguminosae (particularly Papilionoideae) is the predominant host plant taxon [3]. Cultivated legumes, including broad beans (*Vicia faba*) and garden peas (*Pisum sativum*) are among its preferred host plants, and the butterfly is a crop pest in many parts of its range [4]. *Lampides boeticus *is among the approximately three-quarters of butterfly species in the family Lycaenidae that associate with ants as larvae and pupae [5]. The species is facultatively tended by a variety of ants throughout its range, including *Camponotus *spp., *Iridomyrmex *spp., and 'tramp' ant species including *Tapinoma melanocephalum *and the Argentine ant, *Linepithema humile *[6,7].

**Figure 1:**
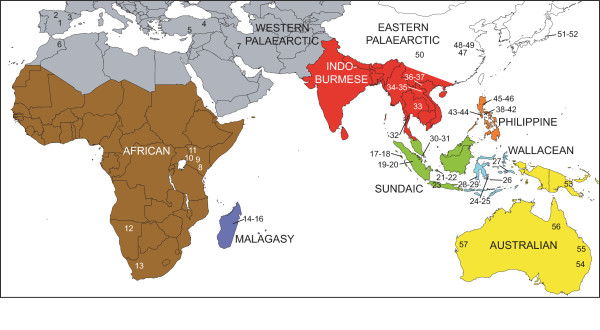
**Map of *Lampides boeticus *collection localities**. Numbers refer to sample information in Table 1. Different colors distinguish labeled biogeographic regions.

We sampled 57 *L. boeticus *from 39 localities on four continents (Fig. 1) to test the hypothesis that this widespread species, as currently circumscribed, consists of more than one genetically distinct taxon. We also used nucleotide sequence data to further examine the genetic structure of this species and analyze the demographic history of the sampled populations.

## Results

### Phylogenetic analyses and node dating

**Figure 2:**
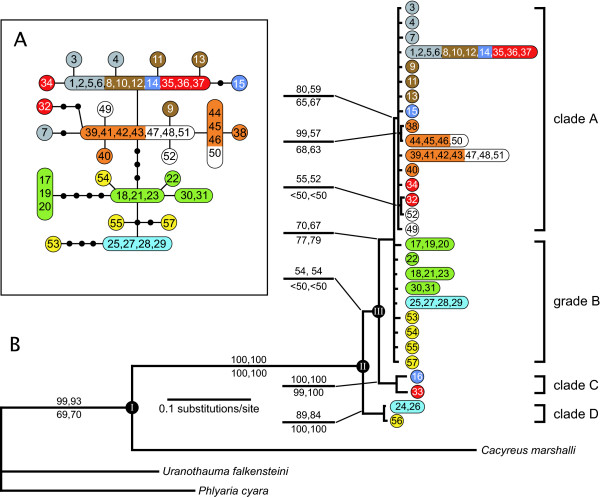
**Relationships among COI+cytB mitochondrial haplotypes of *Lampides boeticus***. Numbers refer to sample information in Table 1; colors denote biogeographic regions designated in Fig. 1. A. Most parsimonious haplotype network of *L. boeticus *constructed with 99% connection limit. Black circles indicate extinct or unsampled haplotypes that differ by one nucleotide substitution from the adjoining haplotype. B. Bayesian consensus tree of *L. boeticus *haplotypes. Numbers above braches indicate Bayesian posterior probability and maximum likelihood bootstrap support, respectively; numbers below indicate parsimony bootstrap symmetric resampling and jackknife support, respectively, for parsimony analyses that resulted in a topologically similar tree. Estimated node ages: I, 6.9 ± 0.6 My; II, 1.5 ± 0.2 My; III, 1.4 ± 0.2 My

Bayesian, maximum likelihood and parsimony phylogenetic analyses arrived at similar phylogenetic hypotheses for the evolutionary history of *L. boeticus *that agreed on all major groupings (Fig. 2B). *Cytochrome c oxidase *subunit I (COI) had 48 variable sites and *cytochrome b *(cytB) had 28, of which 35 and 17 were parsimoniously informative, respectively. Thus, cytB was more variable – 5.35% of nucleotide sites were variable across all samples – than COI, in which 4.10% of nucleotide sites varied. The percentage of parsimoniously informative nucleotide sites was also higher in cytB (3.28% *vs*. 2.87%), as was the number of nucleotide sites with parsimoniously informative non-synonymous substitutions (3 *vs. *0). The parsimony analysis resulted in 1,130 most parsimonious trees with a tree score of 336. The strict consensus of these trees differed with regard to two nodes when compared to the tree obtained in both Bayesian and maximum likelihood analyses (Fig. 2B). *Lampides boeticus *was monophyletic with regard to the three chosen outgroup species. In addition to the divergent genotypes in clades C and D (Fig. 2B), there were two other groups that were supported by Bayesian, maximum likelihood and parsimony analyses. Clade A contained all haplotypes from Africa, Madagascar, the eastern and western Palaearctic, Indo-Burma, and the Philippines not found in the divergent clades C and D. Grade B is a paraphyletic assemblage containing all of the haplotypes from the Sundaland, Wallacean, and Australian regions not found clades C and D. Only samples 26 and 56 from clade D were infected with *Wolbachia *as determined by PCR assay.

Divergence of *Lampides *from its putatively closest relative, *Cacyreus*, occurred in the Miocene approximately 6.9 ± 0.6 Mya (node I; Fig. 2B). Divergence of clade D, containing haplotypes from north Queensland and the Moluccas (node II), occurred in the Pleistocene approximately 1.5 ± 0.2 Mya, and clade A and grade B, containing the majority of haplotypes (node III), diverged approximately 1.4 ± 0.2 Mya (Fig. 2B). However, given the relative paucity of genetic variation and the small magnitude of the difference of the inferred ages of nodes (both of which are likely to increase error), these age estimates should be regarded as approximations.

Pairwise distances among *L. boeticus *COI+cytB haplotypes ranged from 0–2.36% (Table 1), while distances between *L. boeticus *and the outgroup taxa ranged from 7.09–10.78% (data not shown). The relatively low levels of intraspecific sequence divergence among populations are consistent with the hypothesis that *L. boeticus *is a single species with pairwise genetic distances well below the upper ranges of intraspecific divergence estimates found in other lepidopteran species [8,9].

**Table 1:** Collection and voucher information for specimens used in this study

Sample No.	Collecting Locality, elevation	Pairwise Distance from 1	Pairwise Distance from 56	Collection Date	Voucher Code	Voucher Location	COI	cytB
1	Spain: Guadalajara (40°37' N, 3°09' W), 900 m*	0	0.0217	27.VI.2004	102814	RMBR	EU919314	EU919359
2	Spain: Vizcaya (43°20' N, 2°55' W), 200 m*	0	0.0217	2002	102815	RMBR	EU919315	EU919392
3	Spain: Barcelona (41°38' N, 1°59' E), 600 m	0.0006	0.0212	11.VII.1999	MAT-99-T966	MCZ	EU919338	EU919376
4	Turkey: Erzican (39°34' N, 39°56' E), 950 m	0.0006	0.0223	17.VII.2001	VL-01-L275	MCZ	EU919281	EU919373
5	Turkey: 15 km S Çamardi (37°42' N, 35°01' E), 1300 m	0	0.0217	29.VII.2004	RV-04-G379	MCZ	EU919331	EU919385
6	Morocco: Ifrane (34°03' N, 3°46' E), 650 m*	0	0.0217	24.VI.2002	NK-02-A007	MCZ	EU919312	EU919361
7	Iran: Lurestan (33°33' N, 48°50' E), 2100 m	0.0017	0.0202	14.VII.2002	VL-02-X321	MCZ	EU919336	EU919365
8	Kenya: Oloosirkon (1°24' S, 36°49' E), 1700 m	0	0.0217	14.XI.2006	DJM-06-U018	MCZ	EU919301	EU919347
9	Kenya: Rift Valley (1°26' S, 36°49' E), 900 m	0.0011	0.0206	16.VII.2006	DJM-06-U019	MCZ	EU919307	EU919377
10	Kenya: Kakamega Forest (0°20' N, 35°00' E), 1400 m	0	0.0217	8.VI.2006	DJM-06-J963	MCZ	EU919297	EU919384
11	Kenya: Mt. Elgon (1°09' N, 34°33' E), 3000 m*	0.0006	0.0215	12.XII.2005	DJM-06-U008	MCZ	EU919296	EU919362
12	Namibia: Otavi (19°38' S, 17°20' E), 1400 m*	0	0.0217	26.XII.1995	HB-95-Y031	MCZ	EU919308	EU919366
13	South Africa: Northern Cape (31°28' S, 19°46' E), 950 m*	0.0006	0.0208	18.XI.1995	AH-95-Y647	MCZ	EU919322	EU919381
14	Madagascar: Tamatave (18°09' S, 49°20' E), 200 m*	0	0.0217	2004	102816	RMBR	EU919309	EU919344
15	Madagascar: Tamatave (18°09' S, 49°20' E), 200 m*	0.0011	0.0208	2004	102817	RMBR	EU919306	EU919388
16	Madagascar: Tamatave (18°09' S, 49°20' E), 200 m*	0.0202	0.0236	2004	102818	RMBR	EU919305	EU919378
17	Indonesia: North Sumatra (1°14' N, 97°23' E), 100 m*	0.0056	0.0211	N/A	102819	RMBR	EU919303	EU919345
18	Indonesia: North Sumatra (1°14' N, 97°23' E), 100 m*	0.0028	0.0200	N/A	102820	RMBR	EU919334	EU919369
19	Indonesia: West Sumatra (0°33' S, 100°21' E), 150 m	0.0056	0.0211	3.VI.2007	102821	RMBR	EU919284	EU919396
20	Indonesia: West Sumatra (0°33' S, 100°21' E), 150 m	0.0056	0.0211	3.VI.2007	102822	RMBR	EU919317	EU919382
21	Indonesia: Banka-Belitung (2°50' S, 107°55' E), 20 m*	0.0028	0.0200	III.2006	102823	RMBR	EU919329	EU919387
22	Indonesia: Banka-Belitung (2°50' S, 107°55' E), 20 m*	0.0033	0.0206	III.2006	102824	RMBR	EU919286	EU919354
23	Indonesia: Java (6°44' S, 106°33' E), 1000 m	0.0028	0.0200	18.IV.2007	102825	RMBR	EU919325	EU919375
24	Indonesia: SE Sulawesi (4°45' N, 123°55' E), 150 m*	0.0206	0.0011	N/A	102826	RMBR	EU919313	EU919343
25	Indonesia: SE Sulawesi (4°45' N, 123°55' E), 150 m*	0.0039	0.0189	N/A	102827	RMBR	EU919294	EU919348
26	Indonesia: SE Sulawesi (5°00' S, 122°55' E), 300 m*	0.0206	0.0011	N/A	102828	RMBR	EU919339	EU919380
27	Indonesia: Seram/Ambon (3°37' S, 128°10' E), 200 m*	0.0039	0.0189	III.2005	102829	RMBR	EU919321	EU919395
28	Indonesia: South Sulawesi (4°00' S, 120°00' E), 150 m*	0.0039	0.0189	IV.2005	102830	RMBR	EU919300	EU919393
29	Indonesia: South Sulawesi (4°00' S, 120°00' E), 150 m*	0.0039	0.0189	IV.2005	102831	RMBR	EU919320	EU919360
30	Singapore: Kent Ridge (1°17' N, 103°46' E), 50 m	0.0033	0.0206	18.X.2006	102832	RMBR	EU919319	EU919386
31	Singapore: Kent Ridge (1°17' N, 103°46' E), 50 m	0.0033	0.0206	18.X.2006	102833	RMBR	EU919283	EU919352
32	Thailand: Phetchaburi (12°45' N, 99°36' E), 500 m*	0.0022	0.0217	5.VIII.2004	RE-04-C241	MCZ	EU919337	EU919379
33	Thailand: Nakhon Ratchasima (14°50' N, 101°36' E), 300 m	0.0198	0.0232	21.XII.1999	DL-00-Q163	MCZ	EU919324	EU919355
34	Laos: Xam Nuea (20°24' N, 104°05' E), 1200 m*	0.0006	0.0223	17.III.2006	102834	RMBR	EU919302	EU919383
35	Laos: Xam Nuea (20°24' N, 104°05' E), 1200 m*	0	0.0217	23.III.2006	102835	RMBR	EU919333	EU919350
36	Vietnam: Lao Cai (22°15' N, 103°50' E), 1700 m*	0	0.0217	VII.2006	102836	RMBR	EU919327	EU919394
37	Vietnam: Lao Cai (22°15' N, 103°50' E), 1700 m*	0	0.0217	VII.2006	102837	RMBR	EU919299	EU919367
38	Philippines: Marinduque (13°22' N, 121°52' E), 200 m*	0.0017	0.0222	2004	102838	RMBR	EU919298	EU919363
39	Philippines: Marinduque (13°22' N, 121°52' E), 200 m*	0.0006	0.0211	XII.1999	102839	RMBR	EU919335	EU919371
40	Philippines: Marinduque (13°22' N, 121°52' E), 200 m*	0.0011	0.0207	XII.1999	102840	RMBR	EU919311	EU919342
41	Philippines: Marinduque (13°22' N, 121°52' E), 200 m*	0.0006	0.0211	XII.1999	102841	RMBR	EU919280	EU919368
42	Philippines: Marinduque (13°22' N, 121°52' E), 200 m*	0.0006	0.0211	2004	102842	RMBR	EU919316	EU919391
43	Philippines: Oriental Mindoro (11°17' N, 119°40' E), 50 m*	0.0006	0.0211	23.XII.1996	102843	RMBR	EU919291	EU919389
44	Philippines: Oriental Mindoro (11°17' N, 119°40' E), 50 m*	0.0011	0.0217	23.XII.1996	102844	RMBR	EU919285	EU919353
45	Philippines: Quezon (14°2' N, 121°35' E), 200 m*	0.0011	0.0217	1.IX.1996	102845	RMBR	EU919310	EU919357
46	Philippines: Quezon (14°2' N, 121°35' E), 200 m*	0.0011	0.0217	2.IX.1996	102846	RMBR	EU919318	EU919340
47	China: Anhui (30°03' N, 117°34' E), 100 m*	0.0006	0.0211	19.VIII.2002	102847	RMBR	EU919293	EU919341
48	China: Jiangsu (31°20' N, 119°47' E), 100 m*	0.0006	0.0211	1.X.2004	102848	RMBR	EU919288	EU919374
49	China: Jiangsu (31°20' N, 119°47' E), 100 m*	0.0011	0.0218	1.X.2004	102849	RMBR	EU919289	EU919349
50	China: Sichuan (29°20' N, 102°38' E), 1500 m*	0.0011	0.0217	V.2001	102850	RMBR	EU919292	EU919370
51	Japan: Chiba (35°45' N, 140°05' E), 50 m	0.0006	0.0211	1.X.2005	102851	RMBR	EU919330	EU919358
52	Japan: Chiba (35°45' N, 140°05' E), 50 m	0.0017	0.0199	1.X.2005	102852	RMBR	EU919328	EU919372
53	PNG: Morobe (7°20' S, 146°43' E), 1200 m*	0.0061	0.0200	14.V.1999	MFB-99-T893	MCZ	EU919295	EU919390
54	Australia: New South Wales (30°27' S, 151°32' E), 1000 m*	0.0034	0.0208	12.I.1993	NP-93-A001	MCZ	EU919323	EU919364
55	Australia: Queensland (28°16' S, 152°06' E), 500 m*	0.0039	0.0190	5.III.1994	KD-94-R020	MCZ	EU919290	EU919346
56	Australia: Queensland (17°26' S, 145°57' E), 50 m*	0.0217	0	11.VII.1994	KD-94-T055	MCZ	EU919332	EU919356
57	Australia: Western Australia (21°50' S, 114°10' E), 5 m*	0.0045	0.0206	26.X.1997	AAM-97-U361	MCZ	EU919326	EU919351
58†	South Africa: W. Cape, Capetown (33°56' S, 18°30' E), 40 m*	0.1031	0.1054	24.XI.1995	AH-95-Y685	MCZ	EU919304	-
59†	Ghana: Mt. Atewa, Kibi (6°10' N, 2°55' W), 400 m*	0.0838	0.0828	12.VI1996	TL-96-W908	MCZ	EU919282	-
60†	Ghana: Mt. Atewa, Kibi (6°10' N, 1°59' E), 400 m*	0.0831	0.0903	18.IV.1996	TL-96-W917	MCZ	EU919287	-

Specimens 1–57 are *Lampides boeticus*, † = outgroup taxa, 58 = *Cacyreus marshalli*, 59 = *Uranothauma falkensteini*, 60 = *Phlyaria cyara*. Latitude, longitude, and elevation data were estimated from collection locality data using Google Earth http://earth.google.com for taxa marked with an asterisk (*); coordinates and elevations for other taxa were recorded with hand-held GPS. Pairwise distance from 1 = pairwise distance from the most common haplotype, represented by specimen 1; pairwise distance from 56 = pairwise distance from the most divergent haplotype, specimen 56. MCZ = DNA and Tissues Collection of the Museum of Comparative Zoology, Harvard University; RMBR = Cryogenic Collection of the Raffles Museum of Biodiversity Research, National University of Singapore. COI and cytB = GenBank Accession numbers for each specimen

Translated amino acid sequences were invariant within COI, but 16 changes at 8 sites were observed in cytB. McDonald and Kreitman tests found no evidence of natural selection acting on these mitochondrial genes (*P *> 0.20 in all possible pairwise tests).

Perhaps the most striking pattern in the data was the paucity of genetic variation across vast geographic distances. Our analyses showed that *Lampides boeticus *is a widely distributed and apparently panmictic species with little population differentiation. The most common COI+cytB haplotype was shared by specimens from Spain, Turkey, Kenya, Namibia, Madagascar, Laos, and Vietnam, spanning a distance of over 9,000 km or 100 longitudinal degrees on three continents (Figs. 1, 2A, Table 1). Coalescent theory predicts that internal nodes in a gene genealogy will be more common than tip nodes, as these represent older haplotypes. Mutations at different sites within these ancestral haplotypes result in descendent haplotypes that are younger and less common, and appear as multiple 'tips' emanating from the more abundant haplotypes of the internal node [10]. This pattern was evident in our haplotype tree (Fig. 2A). However, several samples were highly divergent from the majority of genetically similar, yet widely distributed haplotypes. These samples could not be connected to the others with a 90% parsimony connection limit in the COI+cytB network (the lowest parsimony value allowed by TCS 1.21; Fig. 2A). These haplotypes, corresponding to clades C and D in the phylogenetic analysis (Fig. 2B), were joined to very different sister haplotypes in the networks of COI and cytB, and with lower parsimony connection limits [see Additional file 1]. In these networks for individual genes, the divergent samples were on relatively long braches, with a haplotype from central Thailand closely related to a sample from Madagascar in clade C. Clade D contained a single sample from north Queensland, Australia, and differed at only two nucleotide sites from a haplotype shared by two samples from the Wallacean islands of Buton and Tomea in the Moluccas to the east of Sulawesi. Interestingly, other samples collected from the same sites in Tomea and Madagascar grouped with the bulk of genetically similar samples (*e.g*., samples 14, 15, and 25 in clade A and grade B, Fig. 2, Additional file 1), indicating substantial genetic diversity within these populations (*e.g*., 2.04% within Madagascar). In the phylogenetic analyses, these lineages appear to have diverged earlier than the more common genotypes (Fig. 2B). It is unlikely that these haplotypes are nuclear copies of the mitochondrial genes (numts), since all sequences could be translated into amino acids with no stop codons. In addition, both genes from the five specimens in clades C and D were amplified and sequenced twice to minimize the probability of human error.

### Demographic and population genetic analyses

Indices of molecular diversity, results of Tajima's *D *and Fu's *F *tests, and output from the mismatch distribution analysis including estimated time since population bottlenecks are provided in Table 2. Grant and Bowen [11] suggested that comparison of *h *and π values within clades can provide information about patterns of past demographic expansion and/or constriction. They categorized numerical values of *h *and π as either high or low, and described situations that may have lead to each of four possible scenarios. In our data set, *h *and π values of COI and cytB from clade A and grade B considered separately or together all fall into category 2, with high *h *(> 0.5) and low π (< 0.005), indicating rapid expansion after a period of low effective population size. All values of Fu's *F *statistic revealed significantly negative deviations from mutation-drift equilibrium (note that, in Fu's *F *analysis, *P *= 0.02 is the threshold value corresponding to α = 0.05) [12]. In addition, Tajima's *D *statistic was significantly negative for COI data from clade A and marginally non-significant for cytB data in the same clade, indicating deviation from neutral evolution and suggestive of demographic expansion.

**Table 2:** Summary of molecular diversity indices and population expansion test statistics

		Molecular Diversity Indices					Tajima's *D*		Fu's *F*		Mismatch Distribution					
	n	No.	S	*k *(var)	***h ***± **SD**	**π **± **SD**	*D*	*P*	*F*	*P*	SSD	*P *(SSD)	τ	**θ**_0_	**θ**_1_	Age in My
**COI**																
**clade A**	35	8	8	0.756 (0.325)	0.556 ± 0.094	0.00066 ± 0.00017	-1.791	**0.0125**	-3.403 × 10^37^	**0.000**	0.00872	0.252	0.781	0.000	99999	0.52
**grade B**	17	7	9	2.132 (1.555)	0.853 ± 0.053	0.00181 ± 0.00032	-0.717	0.264	-23.81	**0.000**	0.0136	0.406	1.129	1.276	105.547	0.75
**clade A + grade B**	52	14	16	2.141 (1.463)	0.782 ± 0.053	0.00187 ± 0.00023	-1.306	0.0801	-26.995	**0.000**	0.00585	0.761	3.457	0.000	3.905	2.30
**cytB**																
**clade A**	35	9	7	0.965 (0.453)	0.692 ± 0.054	0.00185 ± 0.00029	-1.393	0.0725	-30.526	**0.000**	0.108	0.172	1.139	0.004	99999	N/A
**grade B**	17	6	6	1.309 (0.737)	0.779 ± 0.073	0.00236 ± 0.00045	-0.880	0.217	-28.290	**0.000**	0.004	0.675	1.408	0.000	99999	N/A
**clade A + grade B**	52	14	10	2.016 (1.330)	0.830 ± 0.030	0.00387 ± 0.00034	-0.546	0.334	-27.010	**0.000**	0.00150	0.855	2.02	0.533	9.341	N/A

**Figure 3:**
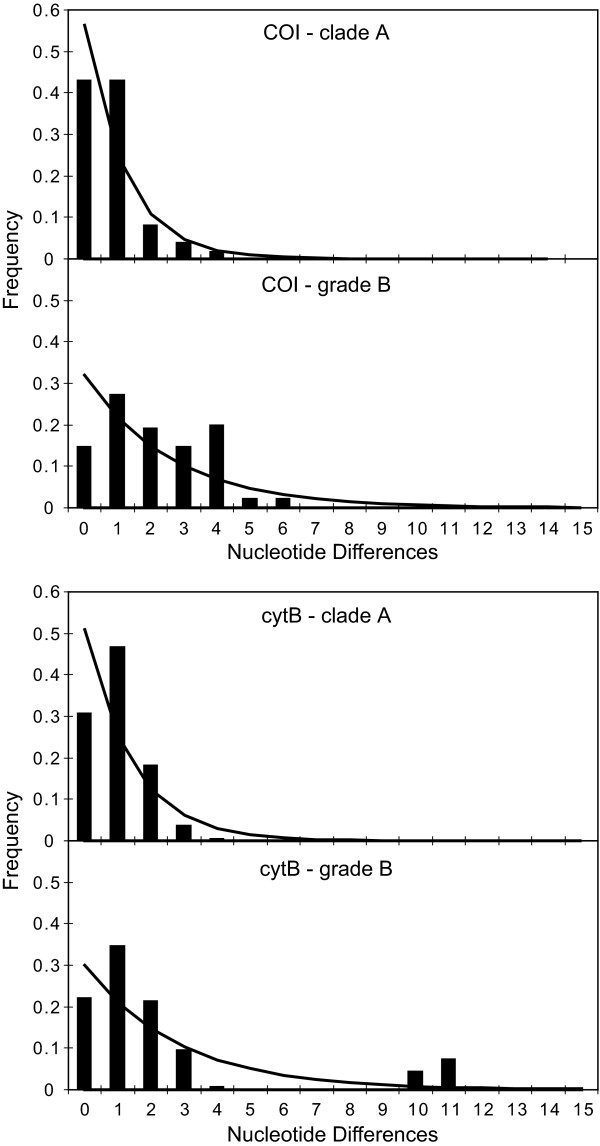
**Mismatch distributions of *Lampides boeticus *lineages clade A and grade B**. Bars indicate observed mismatch differences and lines represent the expected distribution under a sudden population expansion model.

Number of samples (n), number of haplotypes (No.), number of polymorphic nucleotide sites (S), average number of nucleotide differences (*k*) and variance, haplotype diversity (*h*) and standard deviation, and nucleotide diversity (π) and standard deviation calculated for each gene in the two largest and most widespread clades of *Lampides boeticus*. Tajima's *D *and Fu's *F *test statistics with probability values for deviation from neutral evolution and test statistics for mismatch distributions of each gene (Fig. 3), with estimates of time since expansion. Reliable estimates for cytB divergence rates are not available for the calculation of time since expansion. Significant *P *values are bold.

Mismatch distributions are frequency distributions of the number of nucleotide differences in all pairwise comparisons. A population that has experienced sudden exponential growth from an initially small population is expected to have a unimodal mismatch distribution resulting from coalescence of haplotypes to the same bottleneck event [13,14]. In clade A and grade B, deviations of the observed distributions of nucleotide frequencies were not significantly different from those expected under a model of stepwise expansion (Table 2), and visual inspection of the mismatch distributions (Fig. 3) indicate that the unimodal distributions of both genes in clade A are highly suggestive of a bottleneck, while the bimodal distributions of both genes in grade B suggest that the initial population size was larger before the expansion. This difference between clade A and grade B is reflected in differences between the pre- and post-expansion values of θ or the COI data, though this difference is not evident in the θ values calculated from the shorter cytB sequence fragment (Table 2). The estimated age of the bottleneck in clade A is more recent than that of grade B (Table 2), reflecting the more recent divergence of clade A in the estimated phylogeny. The estimated age of the bottleneck for the pair of clades, 2.30 My, predates the estimated divergence time of all extant *L. boeticus *1.5 Mya (Fig. 2B), suggesting methodological discrepancy between the methods used to date divergence times and those used to estimate time since expansion. These differing estimates are no doubt affected by the inflated estimate of time since expansion due to the lack of a value for μ expressed in generations rather than years (see Methods).

Beerli [15] provided evidence from analyses of simulated data that Bayesian methods of population parameter estimation are more likely to provide accurate estimates than maximum likelihood methods. However, our estimations of the parameters θ and *g *using both methods are remarkably concordant. The analyses suggest that the populations constituting grade B are larger than those composing clade A, and that grade B has a higher exponential growth rate than clade A (Table 3).

**Table 3:** Estimation of the effective population size parameter θ and exponential growth rate (*g*) with 95% confidence intervals

	θ	95% CI, θ	*g*	95% CI, *g*
**clade A**	0.00566 (0.00418)	0.00265 – 0.0118 (0.00232 – 0.00868)	926.424 (913.754)	-46.08 – 1017.15 (-164.06 – 1018.09)
**grade B**	0.00711 (0.00711)	0.00181 – 0.0550 (0.00225 – 0.0396)	1274.792 (1286.146)	-412.84 – 5089.14 (-328.14 – 6597.38)
**clade A + grade B**	0.0225 (0.0102)	0.0105 – 0.0720 (0.00635 – 0.0171)	2396.64 (939.65)	1189.87 – 5051.98 (223.90 – 1009.62)

Maximum likelihood estimates are given first; Bayesian estimates are in parentheses.

With the exceptions of *L. boeticus *and the species *Cacyreus marshalli*, which feeds on geraniums and recently underwent a range extension as an invasive species into southern Europe from Africa [16], all species in each of the genera closely related to *Lampides *are confined to the African region, with some species also present in Madagascar. Tracing the most parsimonious reconstruction of biogeographic regions on the given tree indicates that the most recent common ancestor of *L. boeticus *and its sister taxon lived in the African region. However, the optimization for the *L. boeticus *node is ambiguous, with an African origin being only one of several most parsimonious optimizations. The genus *Harpendyreus *was placed in the same section as *Cacyreus *by Eliot [17], implying a close relationship with *Lampides*, but was not sampled in the phylogenetic reconstruction of the Polyommatini. However, *Harpendyreus *is wholly African, and its absence does not affect our inferences.

## Discussion

The phylogeographic structure of *Lampides boeticus *was remarkable in two respects. While most sampled populations had little or no genetic variation across half the planet, a small number of specimens from widely spaced locales were highly divergent from the most common haplotypes, yet relatively similar to each other.

As currently circumscribed, *L. boeticus *appears to be monophyletic with respect to the outgroups included in this analyses, and is nested within a wholly African clade. While we acknowledge the possibility that some extant species may have relictual distributions, it seems plausible – given the number of exclusively African close relatives – that the most recent common ancestor from which *Lampides *and its sister taxon evolved originated in Africa and spread eastward across the Old World. Descendants from early expansion events (clades C and D) appear to have been largely replaced throughout much of the range by descendants from expansion events after one or more recent bottlenecks (clade A and grade B).

The low genetic divergence among most members of this species could be the result of either recent and rapid population expansion after a population bottleneck or selection acting on some portion of the mitochondrial genome. These explanations are not mutually exclusive, but the weight of evidence suggests that most extant populations of *L. boeticus *have descended from expansion after one or more bottlenecks. Results from Fu's *F *and Tajima's *D *tests, analyses of mismatch distributions, and comparison of *h *and π values all suggest rapid expansion after a population bottleneck, with populations in clade A from Africa, Madagascar, the Palaearctic, and the Philippines resulting from a more severe population constriction than populations in grade B from Sundaland, Wallacea, and Australia. Moreover, since sampling was random with respect to genotype, the larger number of post-bottleneck haplotypes in clade A and grade B suggests that these individuals have a selective advantage over individuals from clades C and D, perhaps because they displaced most of the individuals from these basal lineages or because they are more effective dispersers or colonizers. Our data, however, cannot rule out the possibility that genetic drift is responsible for the predominance of haplotypes in clade A and grade B. Since *L. boeticus *is a polyphagous crop pest, human agriculture or commerce may have aided the rapid movement of the species around the globe by inadvertently transporting it with crop plants or produce. However, we suspect this factor has been minimal or geographically restricted, as no individuals from clade A were found in the areas inhabited by individuals from grade B, and *vice versa*.

Inspection of haplotype networks from individual and concatenated genes (Fig. 2A, Additional file 1) further suggest that Australia has been colonized by *L. boeticus *at least three times: once by an ancestor of the genetically divergent individual in clade D (specimen 56), once from a lineage originating in Wallacea (specimens 55 and 57), and at least once by a lineage from Sundaland (specimen 53).

A selective sweep acting on some portion of the mitochondrial genome might also explain the paucity of genetic variation in clade A and grade B. Zink [18] has shown that natural selection on a transmembrane portion of the mitochondrial ND2 gene is responsible for the shallow and unstructured haplotype trees of *Parus montanus *(Aves: Paridae), and similar selective forces on one or more mitochondrial gene(s) could account for the pattern observed in *L. boeticus*. However, MacDonald and Kreitman tests reveal no evidence for selection acting on the genes sequenced for this study.

Infection by *Wolbachia *spp., a group of rickettsial endocellular bacteria that manipulate host reproduction in a variety of ways, can spread rapidly throughout contiguous populations, purging genetic variation of the host species and causing a so-called '*Wolbachia *sweep' [19] capable of reducing haplotype diversity within populations and whole species [20]. However, none of the samples in clade A, grade B, and clade C tested positive for *Wolbachia *infection, though two of the three samples (26 and 56) in clade D were infected. The uninfected specimen in this clade was from a dried specimen of unknown age purchased from a dealer who could not provide the collection date, and may also have been infected, but the *Wolbachia *DNA was too degraded to be amplified in the PCR test employed here.

Four lines of evidence suggest that all *L. boeticus *populations sampled in this study belong to a single, potentially interbreeding species: 1) even the highest *L. boeticus *intraspecific pairwise distance values are within the range of intraspecific variation recorded from other butterfly species; 2) monophyly of all sampled populations is well supported (100% by every measure of clade support); 3) the lack of external morphological differences among populations; and 4) estimated divergence time of all sampled *L. boeticus *genes (approximately 1.5 Mya using a dated node on the phylogeny or 2.3 Mya using coalescence time of COI) is more recent than the estimated divergence of *Lampides *from its putative sister genus *Cacyreus *6.9 Mya. There are many caveats to using pairwise genetic distances and a molecular clock to infer the age of lineage splitting events [21]. These ages must thus remain speculative and, in the case of *L. boeticus*, cannot be confirmed by biogeographic or fossil evidence.

The haplotypes in clades C and D are most likely descended from basal lineages that diverged before the putative population bottleneck(s) that gave rise to clade A and grade B. Descendents of these bottlenecks seem to have largely displaced earlier lineages throughout the species' range. The relatively large number of synapomorphies that unite the haplotypes in clade C and clade D renders convergence unlikely, and the geographical distances between haplotypes in these clades – particularly between the Thai and Malagasy specimens in clade C – makes dispersal an unlikely explanation. The presence of these early-diverging clades, in conjunction with the inferred demographic history of clade A and grade B, suggest a demographic history profoundly affected by the species' propensity to undertake regional migrations [22-24].

The low genetic diversity of *Lampides boeticus *in clade A and grade B across its geographic range is similar to that of several other Old World lepidopteran crop pests. Like *L. boeticus*, the haplotype network of *Helicoverpa armigera *(Noctuidae) has a comparatively long branch, but the cluster of haplotypes at the end of this branch belong to a separate species, *H. zea*, which is morphologically distinguishable only by close examination of the genitalia [25]. The population parameters of θ estimated for clade A and grade B (MLEs of 0.00418 and 0.00711, respectively) overlap with θ values calculated for several other widespread pestiferous Lepidoptera, including *H. armigera *(0.0027 – 0.0099 within western Africa and France/Portugal, respectively) [26] and *Ostrinia nubilalis *(Crambidae; 0.00358 – 0.0315 for different loci within France). Values for the gypsy moth, *Lymantria dispar *(Lymantriidae), are notably lower (0.00129 – 0.00282 for Japan and mainland Asia, respectively) than for *La. boeticus*, but the demographic scenario inferred for *Ly. dispar *is remarkably similar to that of *La. boeticus*: divergent lineages in widespread locales (Japan and India) are thought to represent ancient splitting events from populations spreading throughout Eurasia, with current European populations harboring the most derived haplotypes [27].

## Conclusion

Our analyses suggest that all populations of the butterfly *Lampides boeticus *(Lycaenidae) sampled in this study belong to a single, widespread species with a complex evolutionary history. Phylogenetic estimates suggest that the most recent common ancestor of *L. boeticus *and its sister taxon diverged in Africa in the Miocene approximately 6.9 Mya, but all extant populations coalesce to a most recent common ancestor that lived approximately 2.3 – 1.5 Mya, near the beginning of the Pleistocene. The majority of individuals are descended from expansion events after one or more population bottlenecks, though some populations harbor ancestral polymorphism predating these population constrictions. One lineage found in Northern Australia and the Moluccas may have experienced accelerated differentiation due to infection with the rickettsial endosymbiont *Wolbachia*, which infected at least 2 of 3 sampled individuals in this clade. The proclivity of *L. boeticus *to undergo regional migrations of unknown distance appears to have prevented genetic differentiation due to isolation by distance, and the species' apparent wanderlust has clearly shaped its demographic history, which is marked by expansion and long distance dispersal following periods of small population size.

## Methods

### DNA sequencing and selection of genetic markers

Genomic DNA was extracted from small pieces of abdominal tissue and legs using a CTAB phenol-chloroform extraction protocol, keeping wings and genitalia intact as morphological vouchers. Thirty-four samples were dried, papered specimens up to 11 years old; the bodies of all other specimens were stored in absolute ethanol immediately after collection for molecular study. We chose two rapidly evolving protein-coding mitochondrial genes for our analyses: *cytochrome c oxidase *subunit I (COI) and *cytochrome b *(cytB). The COI gene encompasses the 'Folmer region' used in DNA barcoding, and the cytB gene, located nearly opposite COI on the circular mitochondrial genome [28], is among the most variable protein-encoding genes in insects (R. Meier, unpublished data). Assuming the mitochondrial genome of *L. boeticus *is similar in size and organization to the lycaenid species *Coreana raphaelis *[28], the two genes represent two different regions and approximately 8.5% of the mitochondrial genome.

A 1,220 bp fragment of COI was amplified, typically in two overlapping fragments using LCO1490/Nancy and TN2126/Hobbes primer pairs (Table 4). However, six degraded samples had to be amplified in 3–4 fragments using the following primer pairs: LCO1490/ButterCOI-R2; DanausCOI-F3/ButterCOI-R3; ButterCOI-F4/ButterCOI-R4; and/or ButterCOI-F4/Hobbes (Table 4). The primer pair REVCBJ/REVCB2H successfully amplified a 579 bp region of the cytB gene from all samples. Each sample was also screened for the presence of *Wolbachia*, a maternally inherited bacterial endosymbiont. The primer pair 81F/691R has been used to amplify the *Wolbachia surface protein *(wsp) gene from *Wolbachia *infecting a variety of butterflies and other insects [29-31], and we used these primers in a PCR screen for the presence of *Wolbachia*. Each PCR reaction consisted of 2.5 μl 10× TaKaRa ExTaq buffer with 20 mM MgCl_2_, 1.2 μl of each 10 mM primer, 1 μl 100 mM dNTPs, 0.1 μl TaKaRa ExTaq polymerase, 17 μl H_2_0, and 2 μl template DNA, for a total reaction volume of 25 μl, which was amplified with the following thermal cycler conditions: 3 min at 94°C followed by 35 cycles of 1 min at 94°C, 1 min at 52°C (COI), 54°C (cytB), or 55° (wsp) and 1.5 min at 72°C, and finally 5 min at 72°C. The resulting products were cleaned with Bioline SureClean, labelled with ABI BigDye Terminator 3.1, cleaned with Agencourt CleanSEQ, and sequenced in both directions on an ABI 3130xl DNA analyzer.

**Table 4:** Oligonucleotide primers used in this study

Gene	Primer Name		Primer Sequence	Base Position	Reference
COI	LCO1490	F	GGT CAA CAA ATC ATA AAG ATA TTG G	1501 (38)	[57]
	DanausCOI-F3	F	GTT TGA GCA GTA GGT ATY ACA GC	2029 (566)	this study
	ButterCOI-R2	R	GTA ATT GCY CCA GCT AAA ACW GG	2074 (611)	this study
	TN2126	F	TTG AYC CTG CAG GTG GWG GAG	2133 (670)	R. Eastwood, unpublished
	Nancy	R	CCC GGT AAA ATT AAA ATA TAA ACT TC	2203 (740)	[58]
	ButterCOI-F4	F	GAA TAA TTT ATG CAA TAW TAG CWA TTG G	2296 (833)	this study
	ButterCOI-R3	R	CCA ACT GTA AAT ATA TGA TGR GCT C	2341 (878)	this study
	ButterCOI-R4	R	GAT AAW ACA TAA TGR AAA TGT GCT AC	2599 (1136)	this study
	Hobbes	R	AAA TGT TGN GGR AAA ATG TTA	2743 (1280)	[59]
cytB	REVCB2H	F	TGA GGA CAA ATA TCA TTT TGA GGW	10964 (438)	[60]
	REVCBJ	R	ACT GGT CGA GCT CCA ATT CAT GT	11566 (1040)	[60]
*Wolbachia *wsp	81F	F	TGG TCC AAT AAG TGA TGA AGA AAC	(81)	[61]
	691R	R	AAA AAT TAA ACG CTA CTC CA	(691)	[61]

Direction of amplification is given with reference to the 5' end of the gene. Base position of the primer denotes the position of the last nucleotide on the 3' end of the primer from the 5' end of the *Coreana raphaelis *(Lepidoptera: Lycaenidae) mitochondrial genome and (individual gene sequence) [28]. F = forward primer; R = reverse primer

### Phylogenetic analyses and node dating

Alignment of nucleotide sequences, which contained no indels, was performed with Sequencher 4.6 (Gene Codes Corp.), and data from both genes were concatenated with TaxonDNA 1.5 [32] for subsequent analyses. Replicate COI+cytB sequences were pruned from the dataset so that every haplotype in the phylogenetic analyses was unique. *Cytochrome c oxidase *subunit I (COI) sequences from three closely related butterflies [17] were used to root the ingroup taxa: *Cacyreus marshalli*, *Uranothauma falkensteini*, and *Phlyaria cyara*. Recent molecular phylogenetic investigation of the tribe Polyommatini has shown that these taxa are closely related to *L. boeticus *(N.E. Pierce *et al*., *in prep*.). Analyses performed with and without the inclusion of these sequences in the dataset showed that the absence of cytB sequence data for the outgroup species had no effect on the topology of the phylogenetic estimate for the ingroup.

Parsimony analyses were performed with TNT 1.1 [33]. After increasing the maximum number of saved trees to 3,000, a traditional TBR heuristic search was implemented, performing 1,000 replicates and saving 10 trees per replication, replacing existing trees. To assess confidence in the resulting phylogenetic estimate, the data were subjected to a bootstrap analysis using symmetric resampling [34] implementing a traditional search with 33% change probability (1,000 replicates). The results were summarized as absolute frequencies. In addition, the data were resampled with the jackknife technique using a traditional search with a 36% removal probability replicated 1,000 times.

Bayesian phylogenetic analyses were performed with MrBayes 3.1.2 [35]. MrModeltest 2.2 [36] selected the GTR+I+Γ [37] model for both COI and cytB partitions using Akaike's Information Criterion [38]. Parameter values for the substitution model were estimated from the data and allowed to vary independently between genes. Four Markov chains, one cold and three heated, were run simultaneously for 10 million generations. Trees were sampled every 100^th ^generation. After completion of the analysis, the first 25,000 trees were discarded before a majority-rule consensus tree was calculated from the remaining 75,001 trees. Maximum likelihood analyses were performed with GARLI 0.951 [39] starting from a random tree using the GTR model with all model parameters estimated from the data. The analysis was automatically terminated after the search algorithm progressed 10,000 generations without improving the tree topology by a log likelihood of 0.01 or better. Maximum likelihood bootstrap values were obtained by repeating the analysis 100 times and constructing a majority-rule consensus tree with PAUP* 4.0b10 [40].

The most parsimonious haplotype network of *L. boeticus *was determined with TCS 1.21 [41]. Analyses were run multiple times with varying parsimony connection limits to ascertain the highest limit that would retain each connection. The haplotype networks of each gene and of the two concatenated genes were determined separately. Leading and trailing gaps resulting from declining quality at the ends of sequences and lack of overlap between forward and reverse strands were coded as missing data. These missing data caused only minor problems; only two sequences had missing values for three of the fifty-two parsimoniously variable sites. Anastomoses due to convergence of mutations at two or more nucleotide sites were pruned using the guidelines of Castelloe and Templeton [10] to produce strictly bi- or multifurcating topologies. Nested clade phylogeographic analysis was also performed to evaluate population histories of *L. boeticus*. The results of these analyses are presented in Additional file 1.

In the absence of a robust fossil record and/or vicariance events with which to calibrate divergence times, we estimated divergence times using a molecular clock (*e.g*. [42]). *Cytochrome c oxidase *subunit I (COI) exhibits the least rate homogeneity of any insect mitochondrial gene [43], and age estimations were based only on this gene, which shows an average mean uncorrected pairwise distance of 1.5% per million years (My) across a range of arthropod taxa [44]. Mean uncorrected pairwise distances between all samples on each branch of major bifurcating nodes were calculated with MEGA 4 [45] and divided by 0.015 (1.5%) to obtain a rough estimate of node age.

### Demographic and population genetic analyses

To examine the evolutionary histories of sampled populations, we calculated several population genetic diversity indices that allow inference about demographic history. Identical sequences were not removed from the dataset for these analyses. Given the low levels of genetic variation and the paucity of samples at individual sampling sites, we grouped samples by clade (Fig. 2B) and provide separate analyses for clade A, grade B, and clade A + grade B, which, together, form a monophyletic group. We calculated the following population genetic indices using DnaSP [46]: number of haplotypes, number of variable nucleotide sites (S), haplotype diversity (*h*) and its standard deviation, nucleotide diversity (π) and its standard deviation, and the mean number of pairwise differences (*k*), along with its total variance (including components of stochastic and sampling variance) [47]. The haplotype diversity of a sample indicates the probability that two randomly chosen haplotypes within a sample will be identical [equation 8.5, [48]], while nucleotide diversity calculates the average proportion of nucleotide sites that differ in all pairwise comparisons [47]. In addition, the genetic imprint of rapid population expansion can be detected with Tajima's *D *test [49,50], Fu's *F*-test [12], and by inspection of mismatch distributions, which plot the frequency distribution of observed pairwise differences [13].

Tajima's *D *test, Fu's *F *test, and calculation of observed and expected mismatch values were performed with Arlequin 3.11 [51], along with the moment estimators of the time to expansion (τ), and indices of population sizes before and after the expansion, θ_0 _and θ_1_, respectively [52], which are calculated with a generalized non-linear least-square approach with confidence intervals approximated with 1000 replicates of parametric bootstrapping [53]. To assess the validity of these estimates of demographic expansion, the probability of the sum of square deviations (SSD) between the observed mismatch values and values predicted by the model is approximated by determining the proportion of simulated SSDs that are larger than or equal to the observed SSD [51]. The time since expansion, t, is then calculated by substituting values for τ and μ in the equation τ = 2μt [51], where μ (the mutation rate per site per generation) is 0.75% between ancestor-descendent alleles (*i.e*., half of 1.5%, the average value for arthropod pairwise differences per million years) [44]. Note that τ is expressed in generations, while the value of μ used here is measured in years. Since *L. boeticus *passes through several generations per year, this method of estimating time since expansion is most likely an overestimate.

The relative effective population size parameter θ and exponential growth rate, *g*, as well as their 95% confidence intervals, were estimated using data from both COI and cytB and calculated with LAMARC 2.12b [54]. The two parameters and their confidence intervals were jointly estimated with separate Bayesian and maximum likelihood analyses. Each LAMARC analysis consisted of 3 simultaneous searches with heating temperature adjusted automatically with 15 initial chains sampled every 20 steps with a burn-in of 2000, followed by 6 final chains sampled every 20 steps with a burn-in of 2500. Final most likely estimates (MLEs) were calculated using parameter estimates from three replicated analyses.

To infer the geographical origin of *L. boeticus*, we connected our haplotype tree to a well-sampled, genus-level phylogenetic hypothesis for the tribe Polyommatini based on 4939 bp from seven nuclear and mitochondrial genes. Bayesian and maximum likelihood methods both recovered the following topology for *L. boeticus *and closely related genera: (((*Lampides*, *Cacyreus*) *Actizera*)(*Phlyaria*, *Uranothauma*)) (N.E. Pierce *et al., in prep.*). We coded the biogeographic distributions of each taxon in this phylogeny as a character and traced the most parsimonious reconstruction of biogeographic regions on the tree using MacClade 4.06 [55] to assess the probable region where *L. boeticus *and its most recent common ancestor diverged. MacClade was also used to translate DNA sequences to amino acids for tabulation of non-synonymous substitutions. PAUP* 4.0b10 was used to calculate mean pairwise distances among haplotypes. To determine whether non-synonymous changes might be the result of natural selection on the gene, McDonald and Kreitman tests [56] were performed with DnaSP.

## Abbreviations

CI: confidence interval; COI: *cytochrome c oxidase *subunit I; cytB: *cytochrome b; *GTR (+I+Γ): general time-reversible model (with a proportion of invariable sites and gamma distributed rate variation among sites); m: meters; MLE: most likely estimate; My: million years; Mya: million years ago; ND2: *nicotinamide adenine dinucleotide dehydrogenase *subunit 2; SSD: sum of square deviations; wsp: *Wolbachia *surface protein

## Authors' contributions

DJL coordinated fieldwork, collected specimens, performed the laboratory work and data analyses, and wrote the manuscript. DP assisted with fieldwork, contributed specimens, and helped write the manuscript. NEP assisted in the design of the study, contributed specimens, suggested additional analyses, and helped write the manuscript. RM conceived of the study, participated in the coordination of the study, suggested additional analyses, and helped write the manuscript.

## Supplementary Material

Additional file 1 **Additional information on the GeoDis analysis of our data including haplotype networks of individual and concatenated genes with step clades designated**. Click here for file

## References

[B1] Avise JC Molecular Markers, Natural History and Evolution 2004 2 Sunderland, MA: Sinauer Associates

[B2] Bickford D Lohman DJ Sodhi NS Ng PKL Meier R Winker K Ingram K Das I Cryptic species: a new window on diversity and conservation Trends in Ecology and Evolution 2006 22 148 155 10.1016/j.tree.2006.11.004 17129636

[B3] HOSTS – a Database of the World's Lepidopteran Hostplants http://www.nhm.ac.uk/research-curation/projects/hostplants/

[B4] Mavi GS A critical review on the distribution and host-range of pea blue butterfly, *Lampides boeticus *(Linn.) Journal of Insect Science 1992 5 115 119

[B5] Pierce NE Braby MF Heath A Lohman DJ Mathew J Rand DB Travassos MA The ecology and evolution of ant association in the Lycaenidae (Lepidoptera) Annual Review of Entomology 2002 47 733 771 10.1146/annurev.ento.47.091201.145257 11729090

[B6] Eastwood R Fraser AM Associations between lycaenid butterflies and ants in Australia Australian Journal of Ecology 1999 24 503 537 10.1046/j.1440-169x.1999.01000.x

[B7] Fiedler K Ant associates of Palaearctic lycaenid butterfly larvae (Hymenoptera: Formicidae; Lepidoptera: Lycaenidae) – a review Myrmecologische Nachrichten 2006 9 77 87

[B8] Hebert PDN Cywinska A Ball SL deWaad JR Biological identifications through DNA barcodes Proceedings of the Royal Society (London) B 2003 270 313 321 10.1098/rspb.2002.2218 PMC1691236 12614582

[B9] Zakharov EV Smith CR Lees DC Cameron A Vane-Wright RI Sperling FAH Independent gene phylogenies and morphology demonstrate a Malagasy origin for a wide-ranging group of swallowtail butterflies Evolution 2004 58 2763 2782 15696754 10.1111/j.0014-3820.2004.tb01628.x

[B10] Castelloe J Templeton AR Root probabilities for intraspecific gene trees under neutral coalescent theory Molecular Phylogenetics and Evolution 1994 3 102 113 10.1006/mpev.1994.1013 8075830

[B11] Grant WAS Bowen BW Shallow population histories in deep evolutionary lineages of marine fishes: insights from sardines and anchovies and lessons for conservation Journal of Heredity 1998 89 415 426 10.1093/jhered/89.5.415

[B12] Fu YX Statistical tests of neutrality of mutations against population growth, hitchhiking and background selection Genetics 1997 147 915 925 9335623 10.1093/genetics/147.2.915 PMC1208208

[B13] Slatkin M Hudson RR Pairwise comparisons of mitochondrial DNA sequences in stable and exponentially growing populations Genetics 1991 129 555 562 1743491 10.1093/genetics/129.2.555 PMC1204643

[B14] Rogers AR Harpending H Population growth makes waves in the distribution of pairwise genetic differences Molecular Biology and Evolution 1992 9 552 569 1316531 10.1093/oxfordjournals.molbev.a040727

[B15] Beerli P Comparison of Bayesian and maximum-likelihood inference of population genetic parameters Bioinformatics 2006 22 341 345 10.1093/bioinformatics/bti803 16317072

[B16] Quacchia A Ferracini C Bonelli S Balletto E Alma A Can the Geranium Bronze, *Cacyreus marshalli*, become a threat for European biodiversity? Biodiversity and Conservation 2008 17 1429 1437 10.1007/s10531-008-9350-3

[B17] Eliot JN The higher classification of the Lycaenidae (Lepidoptera): a tentative arrangement Bulletin of the British Museum of Natural History 1973 28 373 505

[B18] Zink RM Natural selection on mitochondrial DNA in *Parus *and its relevance for phylogeographic studies Proceedings of the Royal Society (London) B 2005 272 71 78 10.1098/rspb.2004.2908 PMC1634939 15875572

[B19] Werren JH Biology of *Wolbachia* Annual Review of Entomology 1997 42 587 609 10.1146/annurev.ento.42.1.587 15012323

[B20] Narita S Nomura M Kato Y Fukatsu T Genetic structure of sibling butterfly species affected by *Wolbachia *infection sweep: evolutionary and biogeographical implications Molecular Ecology 2006 15 1095 1108 10.1111/j.1365-294X.2006.02857.x 16599969

[B21] Heads M Dating nodes on molecular phylogenies: a critique of molecular biogeography Cladistics 2005 21 62 78 10.1111/j.1096-0031.2005.00078.x 34892910

[B22] de Vos R Ellis WN Migrating Lepidoptera and rarities in 2001 and recent adventive records Entomologische Berichten 2004 64 138 145

[B23] Dingle H Zalucki MP Rochester WA Season-specific directional movement in migratory Australian butterflies Australian Journal of Entomology 1999 38 323 329 10.1046/j.1440-6055.1999.00117.x

[B24] Corbet AS Pendlebury HM Eliot JN D'Abrera B Butterflies of the Malay Peninsula 1994 4 Kuala Lumpur: Malayan Nature Society

[B25] Behere GT Tay WT Russell DA Heckel DG Appleton BR Kranthi KR Batterham P Mitochondrial DNA analysis of field populations of *Helicoverpa armigera *(Lepidoptera: Noctuidae) and of its relationship to *H. zea* BMC Evolutionary Biology 2007 7 117 10.1186/1471-2148-7-117 17629927 PMC1934911

[B26] Nibouche S Buès R Toubon J-F Poitout S Allozyme polymorphism in the cotton bollworm *Helicoverpa armigera *(Lepidoptera: Noctuidae): comparison of African and European populations Heredity 1998 80 438 445 10.1046/j.1365-2540.1998.00273.x

[B27] Bogdanowicz SM Schaefer PW Harrison RG Mitochondrial DNA variation among worldwide populations of gypsy moths, *Lymantria dispar* Molecular Phylogenetics and Evolution 2000 15 487 495 10.1006/mpev.1999.0744 10860656

[B28] Kim I Lee EM Seol KY Yun EY Lee YB Hwang JS Jin BR The mitochondrial genome of the Korean hairstreak, *Coreana raphaelis *(Lepidoptera: Lycaenidae) Insect Molecular Biology 2006 15 217 225 10.1111/j.1365-2583.2006.00630.x 16640732

[B29] Tagami Y Miura K Distribution and prevalence of *Wolbachia *in Japanese populations of Lepidoptera Insect Molecular Biology 2004 13 359 364 10.1111/j.0962-1075.2004.00492.x 15271207

[B30] Jiggins FM Bentley JK Majerus MEN Hurst GDD How many species are infected with *Wolbachia*? Cryptic sex ratio distorters revealed to be common by intensive sampling Proceedings of the Royal Society (London) B 2001 268 1123 1126 10.1098/rspb.2001.1632 PMC1088716 11375098

[B31] Hiroki M Tagami Y Miura K Kato Y Multiple infection with *Wolbachia *inducing different reproductive manipulations in the butterfly *Eurema hecabe* Proceedings of the Royal Society (London) B 2004 271 1751 1755 10.1098/rspb.2004.2769 PMC1691781 15306297

[B32] Meier R Shiyang K Vaidya G Ng PKL DNA barcoding and taxonomy in Diptera: a tale of high intraspecific variability and low identification success Systematic Biology 2006 55 715 728 10.1080/10635150600969864 17060194

[B33] Goloboff PA Farris JS Nixon KC TNT, a free program for phylogenetic analysis Cladistics 2008 24 774 786 10.1111/j.1096-0031.2008.00217.x

[B34] Goloboff P Farris JS Källersjö M Oxelmann B Ramirez M Szumik C Improvements to resampling measures of group support Cladistics 2003 17 S26 S34 10.1111/j.1096-0031.2001.tb00102.x

[B35] Ronquist F Huelsenbeck JP MrBayes 3: Bayesian phylogenetic inference under mixed models Bioinformatics 2003 19 1572 1574 10.1093/bioinformatics/btg180 12912839

[B36] Nylander JAA MrModeltest v.2.2 2004 Evolutionary Biology Centre, Uppsala University: Program distributed by the author

[B37] Tavaré S Miura RM Some probabilistic and statistical problems in the analysis of DNA sequences Some Mathematical Questions in Biology: DNA Sequence Analysis 1986 Providence, RI: American Mathematical Society 57 86

[B38] Akaike H A new look at the statistical model identification IEEE Transactions on Automatic Control 1974 19 716 723 10.1109/TAC.1974.1100705

[B39] Zwickl DJ Genetic algorithm approaches for the phylogenetic analysis of large biological sequence datasets under the maximum likelihood criterion PhD Thesis 2006 Austin: The University of Texas at Austin

[B40] Swofford DL PAUP*: Phylogenetic Analysis Using Parsimony (*and Other Methods), Version 4.0b10 2002 Sunderland, MA: Sinauer Associates

[B41] Clement M Posada D Crandall KA TCS: a computer program to estimate gene geneologies Molecular Ecology 2000 9 1657 1659 10.1046/j.1365-294x.2000.01020.x 11050560

[B42] Quek S-P Davies SJ Ashton PS Itino T Pierce NE The geography of diversification in mutualistic ants: a gene's-eye view into the Neogene history of Sundaland rain forests Molecular Ecology 2007 16 2045 2062 10.1111/j.1365-294X.2007.03294.x 17498231

[B43] Gaunt MW An insect molecular clock dates the origin of the insects and accords with palaeontological and biogeographic landmarks Molecular Biology and Evolution 2002 19 748 761 11961108 10.1093/oxfordjournals.molbev.a004133

[B44] Quek S-P Davies SJ Itino T Pierce NP Codiversification in an ant-plant mutualism: stem texture and the evolution of host use in *Crematogaster *(Formicidae: Myrmicinae) inhabitants of *Macaranga *(Euphorbiaceae) Evolution 2004 58 554 570 15119439

[B45] Tamura K Dudley J Nei M Kumar S MEGA 4: Molecular Evolutionary genetics Analysis (MEGA) software version 4.0 Molecular Biology and Evolution 2007 24 1596 1599 10.1093/molbev/msm092 17488738

[B46] Rozas J Sanchez-DelBarrio JC Messeguer X Rozas R DnaSP, DNA polymorphism analyses by the coalescent and other methods Bioinformatics 2003 19 2496 2497 10.1093/bioinformatics/btg359 14668244

[B47] Tajima F Evolutionary relationship of DNA sequences in finite populations Genetics 1983 105 437 460 6628982 10.1093/genetics/105.2.437 PMC1202167

[B48] Nei M Molecular Evolutionary Genetics 1987 New York: Columbia University Press

[B49] Tajima F Statistical method for testing the neutral mutation hypothesis by DNA polymorphism Genetics 1989 123 585 595 2513255 10.1093/genetics/123.3.585 PMC1203831

[B50] Tajima F The effect of change in population size on DNA polymorphism Genetics 1989 123 597 601 2599369 10.1093/genetics/123.3.597 PMC1203832

[B51] Excoffier L Laval G Schneider S Arlequin ver. 3.0: An integrated software package for population genetics data analysis Evolutionary Bioinformatics Online 2005 1 47 50 19325852 PMC2658868

[B52] Rogers AR Genetic evidence for a Pleistocene population explosion Evolution 1995 49 608 615 10.2307/2410314 28565146

[B53] Schneider S Excoffier L Estimation of past demographic parameters from the distribution of pairwise differences when the mutation rates vary among sites: application to human mitochondrial DNA Genetics 1999 152 1079 1089 10388826 10.1093/genetics/152.3.1079 PMC1460660

[B54] Kuhner MK LAMARC 2.0: maximum likelihood and Bayesian estimation of population parameters Bioinformatics 2006 22 768 770 10.1093/bioinformatics/btk051 16410317

[B55] Maddison DR Maddison W MacClade 4.06 2003 Sunderland, MA: Sinauer Associates

[B56] McDonald JH Kreitman M Adaptive protein evolution at the Adh locus in Drosophila Nature 1991 351 652 654 10.1038/351652a0 1904993

[B57] Folmer O Black M Hoeh W Lutz R Vrijenhoek R DNA primers for amplification of mitochondrial *cytochrome c oxidase *subunit I from diverse metazoan invertebrates Molecular Marine Biology and Biotechnology 1994 3 294 299 7881515

[B58] Simon C Frati F Beckenbach AT Crespi BJ Liu H Flook P Evolution, weighting, and phylogenetic utility of mitochondrial gene sequences and a compilation of conserved polymerase chain reaction primers Annals of the Entomological Society of America 1994 87 651 701

[B59] Monteiro A Pierce NE Phylogeny of *Bicyclus *(Lepidoptera: Nymphalidae) inferred from *COI*, *COII *and *EF-1alpha *gene sequences Molecular Phylogenetics and Evolution 2001 18 264 281 10.1006/mpev.2000.0872 11161761

[B60] Simmons RB Weller SJ Utility and evolution of *cytochrome b *in insects Molecular Phylogenetics and Evolution 2001 20 196 210 10.1006/mpev.2001.0958 11476629

[B61] Zhou W Rousset F O'Neill S Phylogeny and PCR-based classification of *Wolbachia *strains using *wsp *gene sequences Proceedings of the Royal Society (London) B 1998 265 509 515 10.1098/rspb.1998.0324 PMC1688917 9569669

