# Supplementary material for: Phylogeography and genetic diversity of a widespread Old World butterfly, Lampides boeticus (Lepidoptera: Lycaenidae)
Source: BMC Evol Biol. 2008 Oct 30;8:301. doi: 10.1186/1471-2148-8-301 (PMC2613918; doi:10.1186/1471-2148-8-301)
Supplement: Additional file 1 — Additional information on the GeoDis analysis of our data including haplotype networks of individual and concatenated genes with step clades designated. [file 1471-2148-8-301-S1.pdf]

Additional File

Nested clade phylogeographic analysis

Nested clade phylogeographic analysis (NCPA) has recently come under scrutiny for inferring potentially erroneous population histories [A1-A3], but these claims have been refuted on grounds that the methods used to evaluate NCPA have been flawed [A4, A5]. Templeton and others [A4, A6, A7] suggest that concordant inferences derived from NCPA analyses of multiple, unlinked genetic markers from the same samples provide a method of cross-validating results and reducing type I error. Although we sequenced two linked genes from the non-recombining mitochondrial genome and thus could not cross-validate inferences using multiple loci, we nonetheless performed separate NCPA analyses on our COI and cytB data, expecting that inferences drawn from one gene should mirror inferences from the other.

Most parsimonious haplotype networks were constructed as described in the main text, and nested clades were designated on the resulting networks (Fig. A1). GeoDis 2.5 [A8] was used to examine the relationship between geographic and genetic distances. The number of permutations per test was increased to 10,000 to stabilize the variance associated with the significance levels of each test. The statistical output of the program was interpreted with the most recent version of the inference key (11 November 2005; http://darwin.uvigo.es/software/geodis.html).

Few inferences of population history were consistent between separate NCPA analyses of COI and cytB. For example, interpretation of the GeoDis statistical output suggested that genetic differentiation in the least inclusive nested clade containing the majority of haplotypes found in clade A in the phylogeny could best be explained as restricted gene flow with isolation by distance (IBD) for the COI gene, while analysis of the cytB gene concluded that contiguous range expansion best explained the pattern. These conflicting results between genes were mirrored in analyses considering the nesting clades comparing clade A to grade B: consideration of COI suggested IBD, while analysis of cytB again inferred range expansion. The single point of agreement between the two analyses was the inference that IBD was responsible for geographical partitioning of genetic variation within grade B, which contained most of the haplotypes from the Malay Archipelago, Wallacea, Papua New Guinea, and Australia. However, the lack of samples from Borneo and the paucity of samples from Java (n = 1) might weaken this conclusion, as this poorly sampled area lies between the two

more intensively sampled areas. Analyses of many of the nested clades, including those for haplotypes in clades C and D, failed to reject the null hypothesis of panmixia. Given the lack of concordance between the two separate NCPA analyses performed on the linked mitochondrial genes COI and cytB, we concluded that the method is not robust when applied to the relatively small per-site sample sizes in our data set, and we did not consider the results of these analyses in formulating our conclusions.

Additional References

A1. Knowles LL, Maddison WP: **Statistical phylogeography**. *Molecular Ecology* 2002, **11**:2623-2635.

A2. Petit RJ: **The coup de grâce for the nested clade phylogeographic analysis?** *Molecular Ecology* 2008, **17**:516-518.

A3. Panchal M, Beaumont MA: **The automation and evaluation of nested clade phylogeographic analysis**. *Evolution* 2007, **61**:1466-1480.

A4. Templeton AR: **Statistical phylogeography: methods of evaluating and minimizing inference errors**. *Molecular Ecology* 2004, **13**:789-809.

A5. Templeton AR: **Nested clade analysis: an extensively validated method for strong phylogeographic inference**. *Molecular Ecology* 2008, **17**:1877-1880.

A6. Templeton A: **Out of Africa again and again**. *Nature* 2002, **416**:45-51.

A7. Garrick RC, Dyer RJ, Beheregaray LB, Sunnucks P: **Babies and bathwater: a comment on the premature obituary for nested clade phylogeographical analysis**. *Molecular Ecology* 2008, **17**:1401-1403.

A8. Posada D, Crandall KA, Templeton AR: **GeoDis: a program for the cladistic nested analysis of the geographical distribution of genetic haplotypes**. *Molecular Ecology* 2000, **9**:487-488.

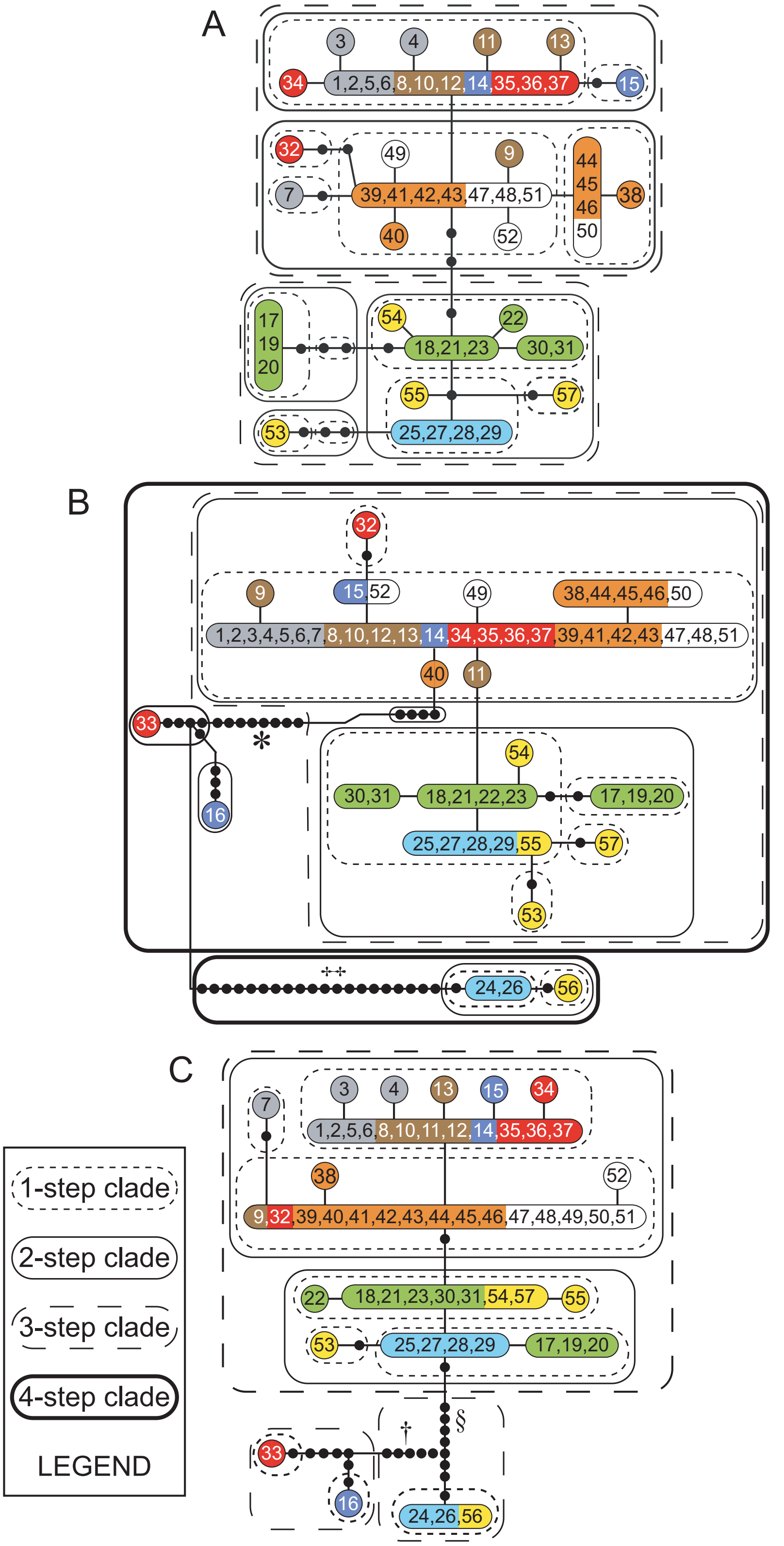

Figure A1
Step clades of the most parsimonious networks of *Lampides boeticus* mitochondrial haplotypes for COI+cytB (A), COI (B) and cytB (C). Black circles indicate extinct or unsampled haplotypes that differ by one nucleotide substitution from the adjoining haplotype. Connection limits of all branches are 99% unless otherwise noted; § denotes 95% connection, * denotes 93% connection limit; † denotes 92% confidence, ‡ denotes 90% connection limit; some step-clades from long branches are excluded for clarity. Numbers refer to specimens listed in Table 1; colors denote biogeographic regions designated in Figure 1.
